# Epigenetic Memory of Early-Life Parental Perturbation: Dopamine Decrease and DNA Methylation Changes in Offspring

**DOI:** 10.1155/2019/1472623

**Published:** 2019-02-19

**Authors:** Laura Bordoni, Cinzia Nasuti, Antonio Di Stefano, Lisa Marinelli, Rosita Gabbianelli

**Affiliations:** ^1^School of Advanced Studies, University of Camerino, 62032 Camerino, Italy; ^2^School of Pharmacy, University of Camerino, 62032 Camerino, Italy; ^3^Dipartimento di Scienze del Farmaco, Università G. d'Annunzio, 66100 Chieti, Italy

## Abstract

Early-life exposure (from postnatal day 6 to postnatal day 21) to permethrin has been associated with long-term development of dopaminergic neurodegeneration in rats. Here, we first investigated if the dopamine decrease observed following early postnatal exposure to permethrin, an oxidative stressor, can impair the dopamine level in the brain of their untreated offspring. Secondly, we evaluated whether this adverse event affects the epigenome of both directly exposed rats (F0) and their untreated offspring (F1). The results show that early-life exposure to the stressor is associated with changes in global DNA methylation and hydroxymethylation in adult age. Furthermore, parental exposure leads to a significant reduction in dopamine level in the offspring (F1) born from parents or just mothers early-life treated (72.72% and 47.35%, respectively). About 2/3 of pups from exposed mothers showed a significant reduction in dopamine level compared to controls. Global DNA methylation and hydroxymethylation impairment was associated with the F1 pups that showed reduced dopamine. This study provides pivotal evidences on intergenerational effects of postnatal exposure to permethrin emphasizing that this xenobiotic can influence the epigenetic memory of early-life parental perturbations disturbing offspring health.

## 1. Introduction

Epigenetic memory of early-life parental perturbations may impact offspring health, because this regulatory mechanism of gene expression may be inherited. Early-life exposure to xenobiotics represents a risk factor associated with epigenetic remodeling due to free radical production [1–4].

Alterations in metabolism due to oxidative stress have a particularly relevant role in the brain, where 5-hydroxymethylcytosine (5hmc), ten-eleven translocation (TET) enzymes, and other global chromatin-modifying proteins have been identified as crucial regulators of both epigenetic and metabolic pathways [5]. Thus, given the scientific evidence, it is of clinical relevance to investigate how epigenetic processes could be involved in the onset of several chronic diseases. Indeed, epigenetic alterations are common elements in several different pathological conditions, including neurodegenerative diseases [6–13].

Since the first 1000 days of life is a window of epigenetic plasticity, the exposure to stressors in this period of life can promote epigenetic remodeling associated with the onset of neurodegeneration later in life [14]. Our previous studies demonstrated that early-life exposure (from postnatal day 6 to postnatal day 21) to oxidative stress induced by the xenobiotic permethrin during brain development promotes behavioral and biochemical changes in the central nervous system. Importantly, we reported that permethrin treatment leads to the development of a progressive Parkinson-like disease in rats, thus identifying this as a validated animal model to study the mechanisms associated with this neurodegenerative disease [15–21]. In particular, our previous studies show that permethrin induces a progressive decrease in dopamine level, from adolescence to old age together with spatial working memory deficits and motor disabilities [16, 18, 20, 22]; dopamine turnover is significantly increased in the animal model of Parkinson, and this catabolic pathway has been associated with early free radical production. However, it is only starting from adult age that age-dependent biomarkers of oxidative stress like decrease in GSH and increase in protein, lipid, and DNA oxidation in the striatum and substantia nigra pars compacta (SNpc) have been observed [15, 16, 18–23]. Furthermore, it has been undoubtedly demonstrated that permethrin promotes oxidative stress in various cell types and tissues isolated from exposed rats (i.e., erythrocytes, leukocytes, heart, and liver striatum) [23–35]. It is important to note that population exposure to the stressor permethrin is habitual because of its wide usage in agriculture for pest control, and its presence in fruits, vegetable, and milk has been significantly demonstrated [36–39]. Residues of permethrin and other members of the pyrethroid family in food are about 25-100 ng/15 g [39]. Furthermore, the presence of the main metabolite 3-phenoxybenzoic acid in people's urine clearly confirms population exposure to this xenobiotic [37, 40]. Due to its lipophilicity, permethrin can be easily absorbed, and we previously demonstrated that it can cross the blood-brain barrier accumulating in the brain and where it remains for a long time even after early-life exposure in rats [19]. Permethrin's ability to promote oxidative stress has also been recognized because its cotreatment with known antioxidants (e.g., vitamin E, vitamin C, coenzyme Q10, tocotrienols, and electrolyzed reduced water) was able to counterbalance the damage induced by its presence [24, 25, 28, 31, 41]. Moreover, we previously reported an increase in DNA methyltransferases, tyrosine hydroxylase, and monomeric and aggregated *α*-synuclein protein levels in adolescent, adult, and old rats exposed to permethrin during brain development [19]. Subsequently, we observed that Nurr1 and global DNA methylation were modified in 33% of untreated offspring, if their parents were exposed to permethrin in their early life [22]. These data indicate that epigenetic remodeling could be associated with nigrostriatal impairments observed in this model [19, 22, 42]. However, no data are available on the role of the mother and the father in the intergenerational inheritance of dopaminergic imbalance nor on the epigenetic mechanisms involved.

Therefore, the first aim of this study was to investigate if early postnatal life exposure (from postnatal day 6 to postnatal day 21) to permethrin of parents (F0) affects dopamine levels in their unexposed offspring (F1). The secondary objective of this study was to investigate the DNA methylation and hydroxymethylation in both parents (F0) and their unexposed offspring (F1) with the aim to identify which epigenetic marks acquired during early life can be transmitted to the next generation. Finally, the third aim was to identify the role of the father and the mother in the intergenerational effect associated with early-life stress exposure.

## 2. Methods

### 2.1. Animal Mating and Treatment: Early-Life Permethrin Exposure (Parents)

**Figure 1:**
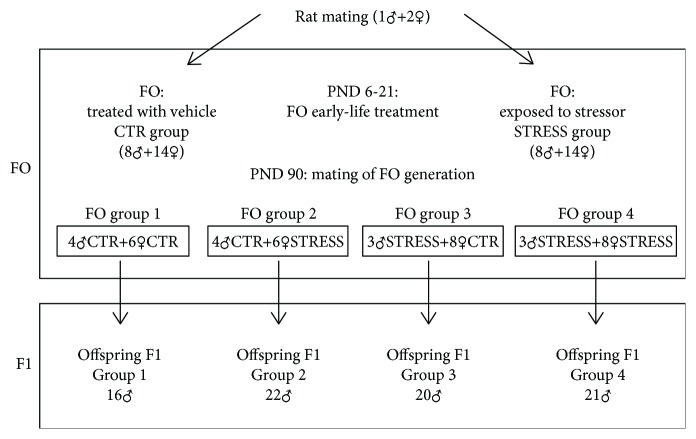
Experimental design, animal mating, and treatment combinations. CTR = control; STRESS = exposed; PND = postnatal days. F1 groups: 1 = control mother and father, 2 = treated mother and control father, 3 = control mother and treated father, and 4 = treated mother and father.

Male and female Wistar rats aged about 90 days weighing 250-270 g were obtained from Charles River (Calco, LC, Italy). Animals were housed, two per cage, in a room with artificial 12 : 12 h light/dark cycle (lights off at 8 : 00 a.m.), at constant temperature (21 ± 5°C) and humidity (45-55%). Food and water were always available in the home cages. Male and female pups born in our laboratory from primiparous dams were assigned to two treatment groups: the animals treated with the stressor permethrin indicated as STRESS and the control, so that each group contained no more than 4 pups (2 males and 2 females) from any litter. Permethrin was solubilized in corn oil and administered to animals by intragastric tube (4 mL/kg) at a dose of 1/50 of LD_50_ corresponding to 34.05 mg/kg (Agency for Toxic Substance and Disease Registry, 2005). The dosage was chosen considering that NOAEL (no observed adverse effect level) for permethrin is 25 mg/kg. The compound was administered daily in the morning from postnatal day (PND) 6 to PND21 [21]. Control group was treated with vehicle (corn oil, 4 mL/kg) on a similar schedule. The volume of solutions was adjusted daily based on body weight of animals. On PND21, the pups were weaned and housed two per cage. At the age of 90 PND, females treated (*n* = 14) or untreated (*n* = 14) with the stressor were mated with males treated (*n* = 6) or untreated (*n* = 8) with the stressor as shown in Figure 1. No siblings were used for mating. F0 generation was then sacrificed at 150 PND; SNpc and the striatum nuclei of each rat were used for DNA methylation and hydroxymethylation assessment.

### 2.2. F1 Generations Born from Different Mating Combinations

The F1 male offspring obtained from different mating combinations (paragraph 2.1) were the main focus of the present study. As reported in Figure 1, the final F1 sample size was a total of 79 male pups divided into 4 groups: *n* = 16 male pups from parents of F0 group 1, *n* = 22 male pups from parents of F0 group 2, *n* = 20 male pups from parents of F0 group 3, and *n* = 21 male pups from parents of F0 group 4. At PND 30, F1 male offspring were sacrificed by exposure to CO_2_. The striatum from each rat was isolated from the brain, immediately placed in liquid nitrogen and stored at -80°C until used for analysis.

All experiments were conducted in accordance with the European Guidelines (Directive 2010/63/EU) for the Care and Use of Laboratory Animals and approved by the local ethic committee.

### 2.3. Dopamine Assessment

Dopamine measurement was performed following the method reported by Gramsbergen and collaborators [43] with slight modifications. Tissues derived from the rat striatum were homogenized with 500 *μ*L of 1 N perchloric acid solution containing 0.02% *w*/*v* sodium metabisulphite and 0.05% *w*/*v* disodium ethylenediaminetetraacetate (Na_2_EDTA). Samples were centrifuged at 4500 × g for 20 min at 4°C. The obtained supernatants were filtered using 0.45 *μ*m filters, collected into vials and stored on ice until analysis. 10 *μ*L of the filtrate was analyzed by HPLC consisting of a Waters 600 pump, a Rheodyne 7295 injector, and an Antec Leyden Decade II detector, operating at +0.75 V. The mobile phase was composed of 0.6% of methanol, 13.61 g/L sodium acetate, 19 mg/L sodium n-octyl sulfate, and 13 mg/mL Na_2_EDTA dissolved in Milli-Q water; the pH was set to 4.1 with glacial acetic acid and degassed with helium. The mobile phase was pumped into a Luna C18 column (250 × 4.6 mm, 5 *μ*m) with a flow rate of 0.6 mL/min. A calibration graph was obtained by preparing various concentrations of dopamine to determine the amount in each striatum sample [44, 45]. Final values are expressed as ng/mg tissue.

### 2.4. DNA Extraction and Global DNA Methylation and Hydroxymethylation Assessment

To isolate sperm DNA of adult rat of the F0 generation, cauda sperms of each father were washed twice with PBS, resuspended in 1.0 mL lysis buffer containing 20 mM Tris (pH 8), 10 mM dithiothreitol, 150 mM NaCl, 10 mM EDTA (pH 8), and 1% SDS, and incubated for 20 h at 37°C [44]. The DNA was extracted from the lysed tissue using DNAzol reagent (Thermo Fisher Scientific Inc., Waltham, MA, USA) following the manufacturer's instructions. Subsequently, 5mC DNA ELISA Kit (Zymo Research s.r.l., Irvine, CA, USA) was used to evaluate differences in global 5mC in sperm DNA from treated fathers with respect to controls.

SNpc and the striatum nuclei of each rat of F0 and F1 generations were also used to extract genomic DNA using DNAzol (Thermo Fisher Scientific Inc., Waltham, MA, USA), according to the manufacturer's instructions. For SNpc and the striatum nuclei DNA analysis, four subgroups for each treatment were set up, basing the grouping on quartiles of dopamine data distribution. 100 ng of DNA for each subgroup sample was then used to evaluate global 5mC and 5hmC levels using, respectively, the 5mC DNA ELISA Kit™ and the Quest 5hmC™ DNA ELISA Kit (Zymo Research s.r.l., Irvine, CA, USA). Results are presented as percentage of total CpG of rat genome. Each sample was analyzed in duplicate following the manufacturer's instructions.

### 2.5. Statistical Analyses

Throughout the study, data are presented as mean ± SD. To calculate the adequate sample size, we performed a power analysis based on effect size observed in our preliminary data [22]. Specifically, the computed effect size (*δ*) of 0.994 was used to perform an a priori power analysis (*α* = 0.05, 1-*β* = 0.80) which showed that the sample size required for each group was 15. Power analysis was performed using G∗Power version 3.1.9.2 (Dusseldorf, Germany).

The Shapiro-Wilk test was used to evaluate the normality of distributions. The Kruskal-Wallis or ANOVA and post hoc analysis with Tukey correction were used, respectively, as parametric or nonparametric tests for multiple comparisons. The Student *t*-test was used to compare means between two groups. Correlation between variables was measured by calculating linear regression and Spearman's rho. Two-tailed *p* values for all the mentioned tests are reported. Statistical analysis and graphs were performed using SPSS [46] or R Studio [47].

## 3. Results

### 3.1. Global DNA Methylation and Hydroxymethylation in Parental F0 Generation

**Figure 2:**
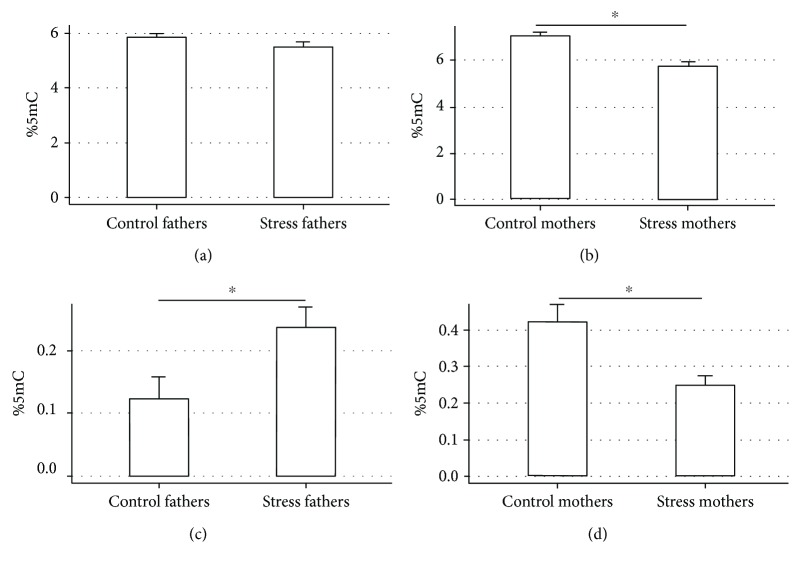
Global methylation (a, b) and hydroxymethylation (c, d) of DNA extracted from the striatum nucleus and SNpc in parental F0 generation. *p* = 0.021 (b), *p* = 0.049 (c), and *p* = 0.047 (d).

Global DNA methylation in the striatum and SNpc in the parental generation was not significantly reduced in males (*p* > 0.05) (Figure 2(a)), while the reduction was significant in female rats treated in early life to stressor (STRESS) with respect to the control females (*p* = 0.021) (Figure 2(b)). Interestingly, the early-life exposure to the stressor induced a significant increase in global 5hmC in males (*p* = 0.049) (Figure 2(c)) and a relative reduction of this epigenetic signature in females (*p* = 0.047) in this tissue (Figure 2(d)). No significant differences could be observed for sperm DNA methylation between treated fathers and controls in this study (data not shown).

### 3.2. Dopamine Levels in F1 Generation

**Figure 3:**
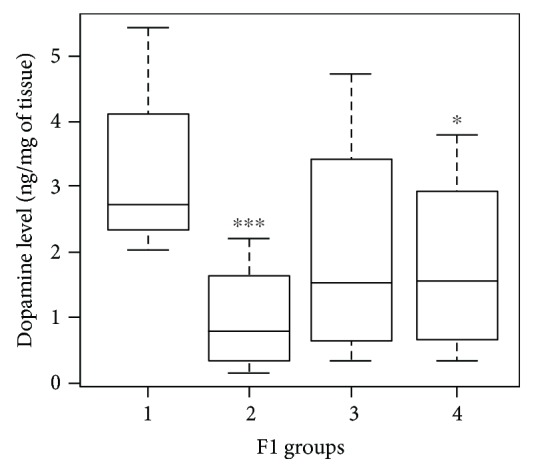
Dopamine level variation with respect to controls (group 1) in the striatum nucleus and SNpc in F1 groups (1 vs. 2, *p* < 0.001; 1 vs. 4, *p* = 0.035). F1 groups: 1 = control mother and father, 2 = treated mother and control father, 3 = control mother and treated father, and 4 = treated mother and father.

The analyzed F1 generation was composed of 79 rats subdivided as follows: 16 rats from both control parents (group 1), 22 rats from treated mothers and control fathers (group 2), 20 rats from control mothers and treated fathers (group 3), and 21 rats originated from both mothers and fathers treated with the stressor (group 4). The mean dopamine level assessed was 1.937 ng/mg (±1.666) throughout the entire F1 generation, with a minimum of 0.02 ng/mg and a maximum of 7.99 ng/mg. Multiple comparisons showed significant differences between dopamine levels measured in the offspring originated from control parents (group 1) with respect to the F1 obtained from both treated parents (group 4) (*p* = 0.035) and treated mother/control father (group 2) (*p* < 0.001). Reductions of 47.35% (group 4) and 72.72% (group 2) in dopamine levels were observed compared to the control group. The reduction induced in F1 groups 2 and 4 did not significantly differ from each other (*p* > 0.05). No relevant changes in dopamine level were observed for the group with control mothers/STRESS fathers (group 3) with respect to the other groups (*p* > 0.05). These results (Figure 3), together with no alteration observed in parental sperm DNA methylation (data not shown), suggest a maternal transmission of the altered phenotype to the F1 generation.

**Figure 4:**
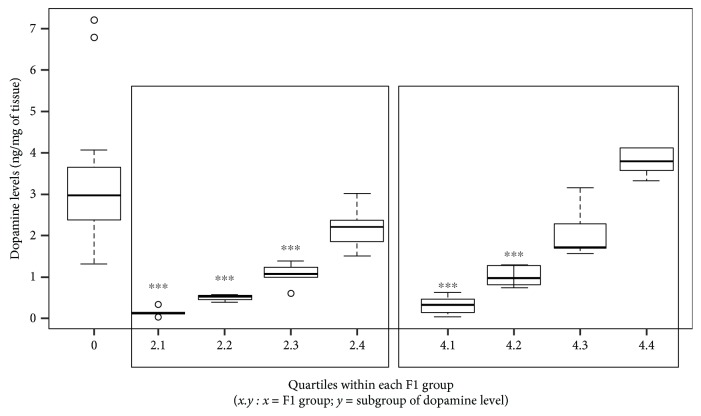
Different levels of dopamine reduction measured in the striatum nucleus and SNpc within the F1 groups 2 and 4 with respect to controls (group 1) (1 vs. 2.1, *p* < 0.001; 1 vs. 2.2, *p* < 0.001; 1 vs. 2.3, *p* < 0.001; 1 vs. 4.1, *p* < 0.001; and 1 vs. 4.2, *p* < 0.001). F1 groups: 1 = control mother and father, 2 = treated mother and control father, 3 = control mother and treated father, and 4 = treated mother and father. Each subgroup represents a quartile of the dopamine distribution within the parental treatment group.

Furthermore, by dividing the groups that significantly differ from the controls based on the quartile of the dopamine distribution observed, we noticed that the reduction in dopamine was not homogenous within each treatment group (Figure 4). About 64% of the rats from treatment groups 2 and 4 actually showed a significant reduction if compared to controls (1 vs. 2.1, *p* < 0.001; 1 vs. 2.2, *p* < 0.001; 1 vs. 2.3, *p* < 0.001; 1 vs. 4.1, *p* < 0.001; and 1 vs. 4.2, *p* < 0.001). These results demonstrate that a significant variability in the inheritance of the altered phenotype exists.

### 3.3. Global DNA Methylation and Hydroxymethylation in F1 Generation

**Figure 5:**
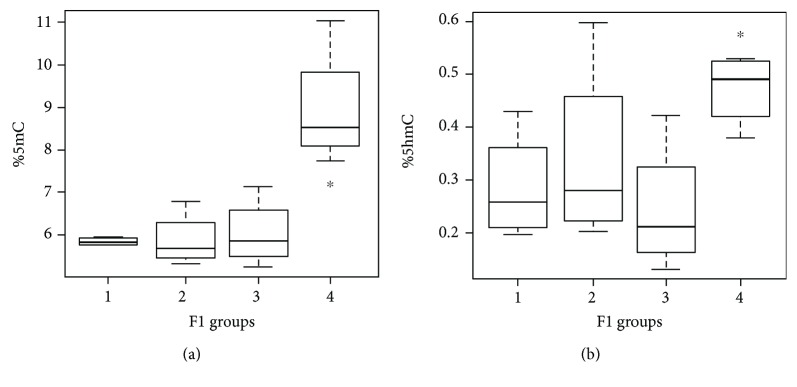
Methylation (a) and hydroxymethylation (b) of DNA extracted from the striatum nucleus and SNpc in F1 generation divided for parental treatments (a) 1 vs. 4 (*p* = 0.033) and (b) 4 vs. 3 (*p* = 0.021); F1 groups: 1 = control mother and father, 2 = treated mother and control father, 3 = control mother and treated father, and 4 = treated mother and father.

Analysis in the F1 generation showed significant differences in global DNA methylation in the striatum and SNpc of rats from differentially treated parents. Specifically, an increase in global 5mC for the group originated from both treated parents (group 4) was detected compared to the others (1 vs. 4, *p* = 0.033; 2 vs. 4, *p* = 0.021; and 3 vs. 4, *p* = 0.021) (Figure 5(a)). Moreover, a similar but less marked trend was observed for 5hmC (Figure 5(b)). In particular, 5hmC in F1 group 4 differed significantly from group 3 (*p* = 0.021) and showed a similar but not significant trend with respect to group 1 (*p* = 0.057) (Figure 5(b)), suggesting that 5hmC levels can be influenced by parental treatment as well.

**Figure 6:**
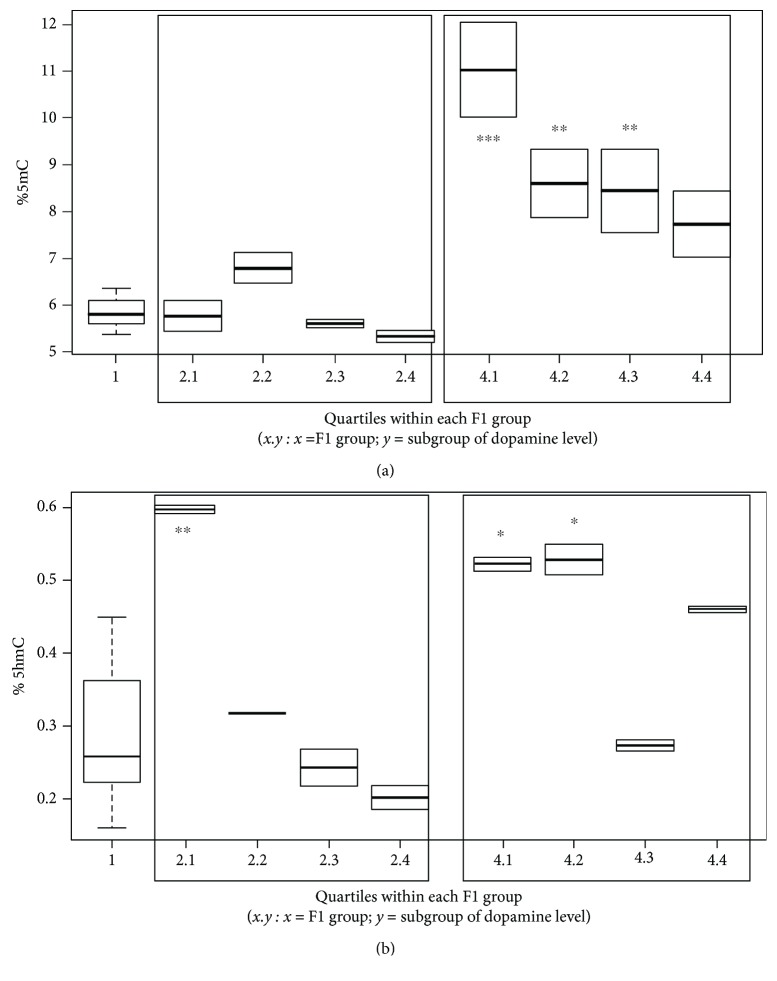
Methylation (a) and hydroxymethylation (b) of DNA extracted from the striatum nucleus and SNpc in subgroups originated by different dopamine reduction levels within F1 groups 2 and 4. (a) 1 vs. 4.1, *p* < 0.001; 1 vs. 4.2, *p* = 0.003; and 1 vs. 4.3, *p* = 0.005 and (b) 1 vs. 2.1, *p* = 0.001; 1 vs. 4.1, *p* = 0.014; and 1 vs. 4.2, *p* = 0.011.

Considering the previously observed variance for the altered phenotype inheritance in terms of dopamine production impairment (Figure 4), we analyzed the variation of 5mC and 5hmC within each parental treatment group that had significantly decreased dopamine levels, according to the quartile of dopamine reduction observed. We found that, as observed for the phenotype, the transmission of the epigenotype to the F1 generation does not occur homogeneously. Specifically, the increase in 5mC occurs in 3 of 4 quartiles within the offspring generated by both treated parents (group 4) (1 vs. 4.1, *p* < 0.001; 1 vs. 4.2, *p* = 0.003; and 1 vs. 4.3, *p* = 0.005), while no significant differences in any of the subgroups for the F1 group 2 were observed (*p* > 0.05) (Figure 6(a)).

These data suggest that 5mC is related to the parental treatment more than to the dopaminergic level. Concerning with 5hmC, only the subgroups characterized by the lowest levels of dopamine (2.1, 4.1, and 4.2 subgroups) showed an increase in this epigenetic mark compared to controls (1 vs. 2.1, *p* = 0.001; 1 vs. 4.1, *p* = 0.014; and 1 vs. 4.2, *p* = 0.011), suggesting a potential correlation between 5hmC and dopamine levels (Figure 6(b)). To corroborate this hypothesis, we tested the correlation between 5hmC and dopamine levels, and we observed a drift in the linear regression test (*p* = 0.1076, *R* ^2^ = 0.1743) (Figure 1(a) supplementary materials) and a *ρ* for the trend for the Spearman's correlation between these two variables (*p* = 0.072, Spearman's rho). Although these results are not significant, a weak relationship between 5hmC and dopamine levels may be inferred. On the other hand, a significant correlation between the increase in 5mC and that in 5hmC was detected (*p* = 0.008, Spearman's rho) (*p* = 0.024, *R* ^2^ = 0.312) (Figure 1(b) supplementary materials).  Figure 2 of supplementary materials shows how 5hmC and 5mC impairments identify the pups originated by both treated parents (group 4), which differ from the others in their epigenetic profile.

## 4. Discussion

DNA methylation is a pivotal epigenetic mark exerting a crucial role in a variety of cellular processes (i.e., gene expression regulation, genomic imprinting, silencing of transposable elements, and X chromosome inactivation) and that specifically plays important roles in mammalian neuronal system [48, 49]. Recent discoveries have demonstrated that 5hmC, which represents an oxidized derivative of 5mC produced by the process of active DNA demethylation, plays an essential role in neuronal tissues. Of note is that 5hmC is not equally distributed across different tissues: it is approximately 10-fold more abundant in neurons than in other cells and particularly enriched in the vicinity of genes with synapse-related functions [48, 50]. Moreover, the amount of 5hmC in the brain increases in an age-dependent manner, suggesting that 5hmC does not just mediate the demethylation process [48, 51–53], but might have a role as an important and stable epigenetic marker in the brain. In support of these evidences, several studies have indicated the dysregulation of 5hmC, as well as of 5mC, as potentially being involved in multiple diseases including neurodevelopmental disorders (i.e., Rett syndrome, autism) and neurodegenerative diseases (i.e., Huntington's disease, Alzheimer's disease, and Parkinson disease) [48, 54–58].

Recent data revealed that global DNA methylation and hydroxymethylation in the striatum nucleus and SNpc of adolescent rats are increased following early-life permethrin treatment [42]. Additionally, preliminary data showed that permethrin-treated female rats have decreased levels of 5mC at adult age, and that this same 5mC reduction could be observed in the adolescent F1 generation, both in the male and female progenies [23].

Following these evidences, in this study, we demonstrated that DNA methylation decreases in directly exposed female rats at adult age, whereas this reduction is not likewise relevant in male adult rats. These results suggest that DNA methylation may increase at the early stage of the damage and decrease later in life (Figure 3 supplementary materials). Concurrently, even if previous researches suggested potential impairment of sperm DNA methylation of rats exposed to this stressor [59–61], in the present study, we did not observe any significant variation in this parameter in early-life-treated rats. However, this paper highlights that the treatment was able to affect not just DNA methylation but also DNA hydroxymethylation in the striatum nucleus and SNpc of adult rats, and again a sex-dependent effect was observed: while adult females displayed a reduction in 5hmC, male rats showed an increase in this epigenetic mark. These data are not completely surprising considering that sex-dependent variations in 5mC and 5hmC have already been reported [62–64]. The intricate relationship between 5mC and 5hmC [65] becomes even more complex if we consider that not just physiological processes but also environmental stimuli could modulate it [1, 62]. Since permethrin is a well-known oxidative stressor [15–31], it is reasonable to assume that this kind of xenobiotic can interfere with normal 5mC/5hmC homeostasis and that different responses could occur in different genders.

An important innovative aspect demonstrated in this study is that not just epigenetic marks acquired during pregnancy but also those established during postnatal early-life can be inherited. Specifically, exposure to the stressor in postnatal early-life (from PND6 to PND21) of the parental generation leads to a significant reduction in dopamine in their offspring, if both parents or just the mothers are treated (dopamine reduction 47.35% and 72.72% vs. control, respectively). Furthermore, not just the phenotype, represented by an impairment in the dopaminergic pathway, but also the epigenotype, in terms of global DNA methylation and hydroxymethylation, are associated with the altered F1 phenotype.

How epigenetic inheritance is transmitted is still unclear [62–66]. Previous investigations demonstrated that 5mC signatures developmentally acquired will be erased in the early embryo and in the germline during a process named epigenetic reprogramming [65]. Nevertheless, recent genome-wide DNA methylation profiling demonstrated that, if germline reprogramming partially fails, a certain number of loci can escape reprogramming, indeed representing the prime candidates for transgenerational epigenetic inheritance in mammals [63, 65–67]. On the other hand, no evidences on the possibility that 5hmC can be directly inherited have been discussed until now. 5hmC accumulates inside the brains during the life span, from neural progenitors through young neurons in the fetal brain, and further during aging of the brain after birth [68]. Additional studies are necessary to establish if 5hmC variation is a consequence of the inherited DNA methylation impairment or if other molecular pathways are indirectly involved in the alteration of this epigenetic mark.

Moreover, a key result of this study is that maternal exposure to the stressor permethrin was the most effective parameter for reducing dopamine levels (72.72% vs. control) in their respective new born pups; thus, a maternal transmission not due to an exposure during pregnancy, but to an epigenetic memory of an early-life perturbation, can be theorized (Figures 2 and 4 supplementary materials).

Several examples of epigenetic inheritance linked to environmental exposures which are heritable through the female germline have recently been described [69, 70], and different biological processes have been suggested to explain these phenomena involving intergenerational or transgenerational effects [71]. Uncertainties on this topic are still present [64] probably because the majority of studies have focalized on epigenetic inheritance through treatments during gestation and have identified heritable epigenetic changes based on differences observed between two populations without assessing if a particular individual inherited the epigenetic state of his/her parent [62]. In light of these drawbacks, the present study is important to improve the knowledge on the complex interaction between the environment and the epigenome in the context of neurodegeneration.

This study has two important limitations. First of all, global DNA methylation and hydroxymethylation have been measured, which provide a general estimation of epigenome perturbations without targeting the genomic regions involved. Moreover, since epigenetics is extremely cell-specific, and given the cellular heterogeneity and differences in cell type composition across brain regions, a reduction of bias should have been provided by analysis of the epigenome at single-cell resolution. Nevertheless, this study is based on a powerful animal model characterized by a progressive neurodegenerative disease onset, where damages are slowly induced by only 15 days of low dosage exposure to a stressor after birth during brain development. This represents the best condition to study epigenetic modifications (slow to occur) and best mimics real effects of subtle exposure to other environmental stressors [15–35].

In conclusion, since the F1 generation did not receive any permethrin, the impairments observed in DNA methylation and hydroxymethylation, together with reduction in dopamine levels in the F1 generation, have to be associated with parental early-life exposure to permethrin. This confirms that epigenetics is involved in the induction of this intergenerational impairment of the dopaminergic pathway. Not just epigenetic alterations established during pregnancy but also the epigenetic memory of early-life maternal events can impact offspring health, as observed in this study.

Further researches able to clarify the mechanisms involved in the intergenerational inheritance of early-life environmentally induced epigenotype would provide the basis to identify early determinants of late-onset diseases, helping to reduce the burden of neurodegenerative pathologies that characterize modern society.

## Data Availability

The animal mating and treatment, dopamine assessment, DNA extraction and global DNA methylation and hydroxymethylation assessment, and statistical analyses data used to support the findings of this study are included within the article.

## Abbreviations

SNpc: Substantia nigra pars compacta
5mC: 5-Methylcytosine
5hmc: 5-Hydroxymethylcytosine
PND: Postnatal day
TET: Ten-eleven translocation.

## Supplementary Materials

Supplementary Materials Supplementary materials clarify data described in the main document and help to understand their relevance as a part of a major project which investigates neurotoxic effects of early-life exposure to PERM. In particular, Figure 1 of supplementary materials shows row data describing correlations calculated between dopamine, 5mC and 5hmC, whose relevance has been discussed in the paper. Similarly, Figure 2 clearly shows how 5mC and 5hmC are able to describe analyzed F1 groups and in particular to generate a cluster of those originated from both treated parents with respect to the others. On the other hand, Figure 3 integrates data described in this paper with others previously collected on the same animal model. Finally, Figure 4 of supplementary material provides a graphical overview of all the effects (dopamine reduction, 5mC and 5hmC alterations) of parental treatments in their F1 offspring. In this context, supplemental data help the reader to understand the major data and to contextualize them in a broader prospective.
