# Supplementary material for: Epigenetic Memory of Early-Life Parental Perturbation: Dopamine Decrease and DNA Methylation Changes in Offspring
Source: Oxid Med Cell Longev. 2019 Feb 19;2019:1472623. doi: 10.1155/2019/1472623 (PMC6399534; doi:10.1155/2019/1472623)
Supplement: Supplementary Materials — clarify data described in the main document and help to understand their relevance as a part of a major project which investigates neurotoxic effects of early-life exposure to PERM. In particular, Figure 1 of supplementary materials shows row data describing correlations calculated between dopamine, 5mC and 5hmC, whose relevance has been discussed in the paper. Similarly, Figure 2 clearly shows how 5mC and 5hmC are able to describe analyzed F1 groups and in particular to generate a cluster of those originated from both treated parents with respect to the others. On the other hand, Figure 3 integrates data described in this paper with others previously collected on the same animal model. Finally, Figure 4 of supplementary material provides a graphical overview of all the effects (dopamine reduction, 5mC and 5hmC alterations) of parental treatments in their F1 offspring. In this context, supplemental data help the reader to understand the major data and to contextualize them in a broader prospective. [file 1472623.f1.docx]

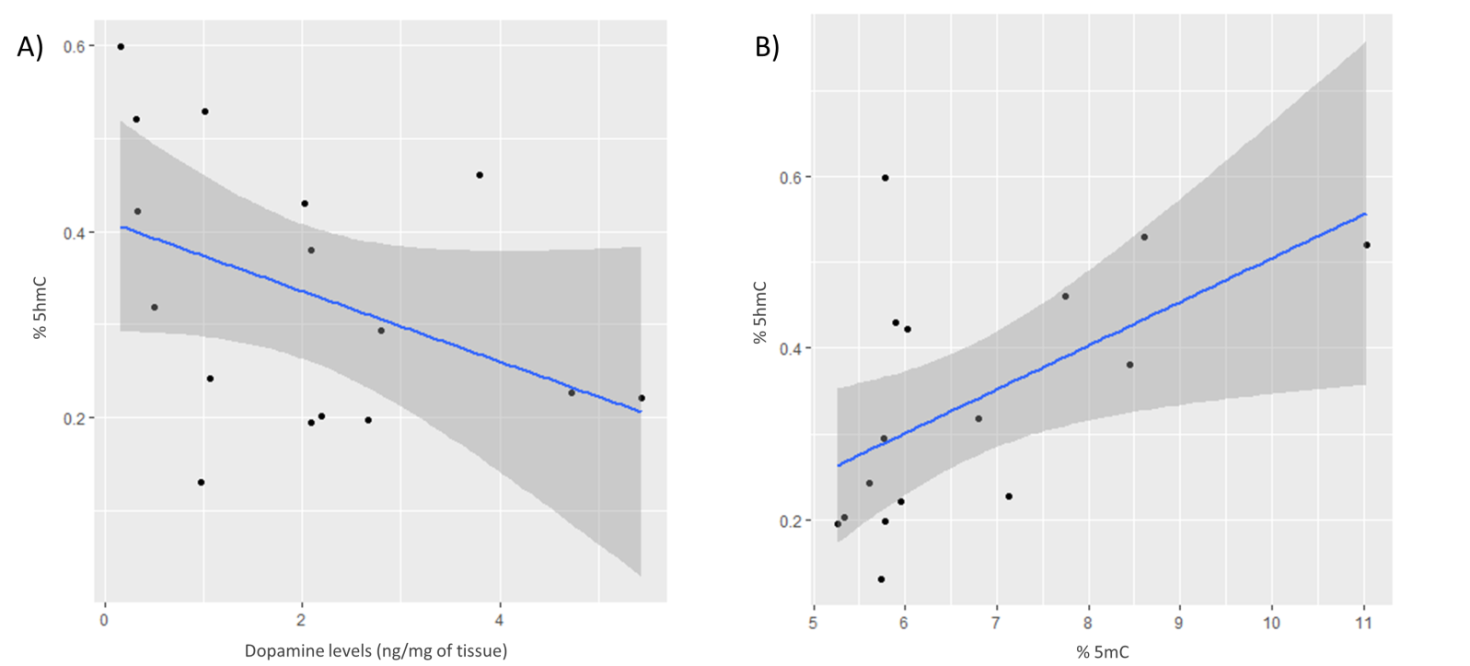


**Figure 1 supplementary materials.** Linear regression plot testing correlation between % 5hmC and dopamine levels (A) and % 5hmC and % 5mC (B).


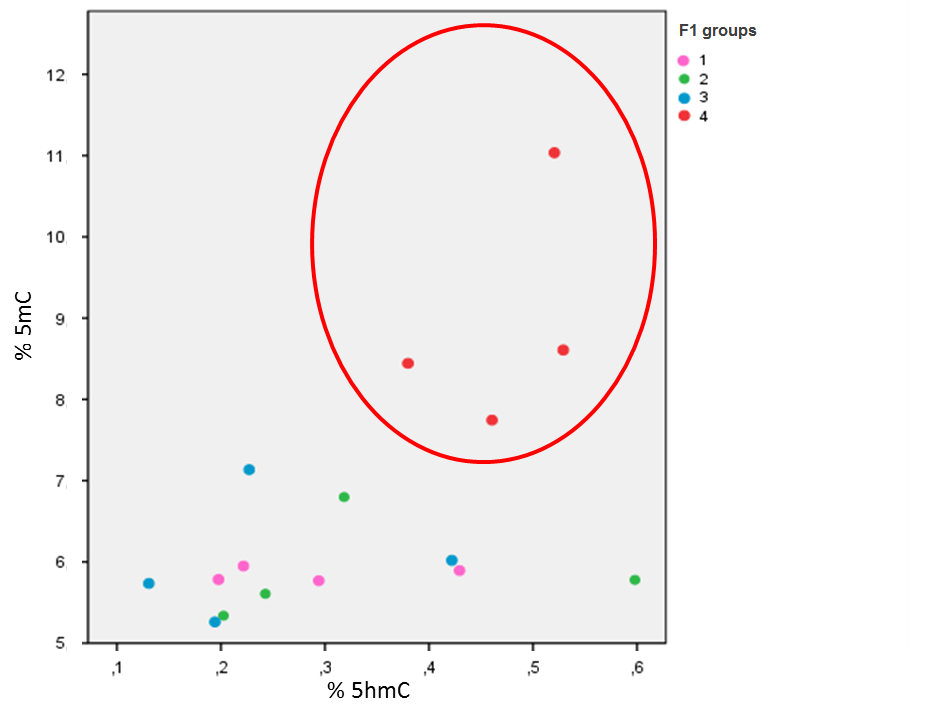


**Figure 2 supplementary materials.** Plot representing dopamine quartile subgroups for each parental treatment respect to % 5mC and % 5hmc. F1 groups are represented in different colors: 1= control mother and father~~;~~ 2 = treated mother, control father; 3= control mother, treated father; 4= treated mother and father.

**
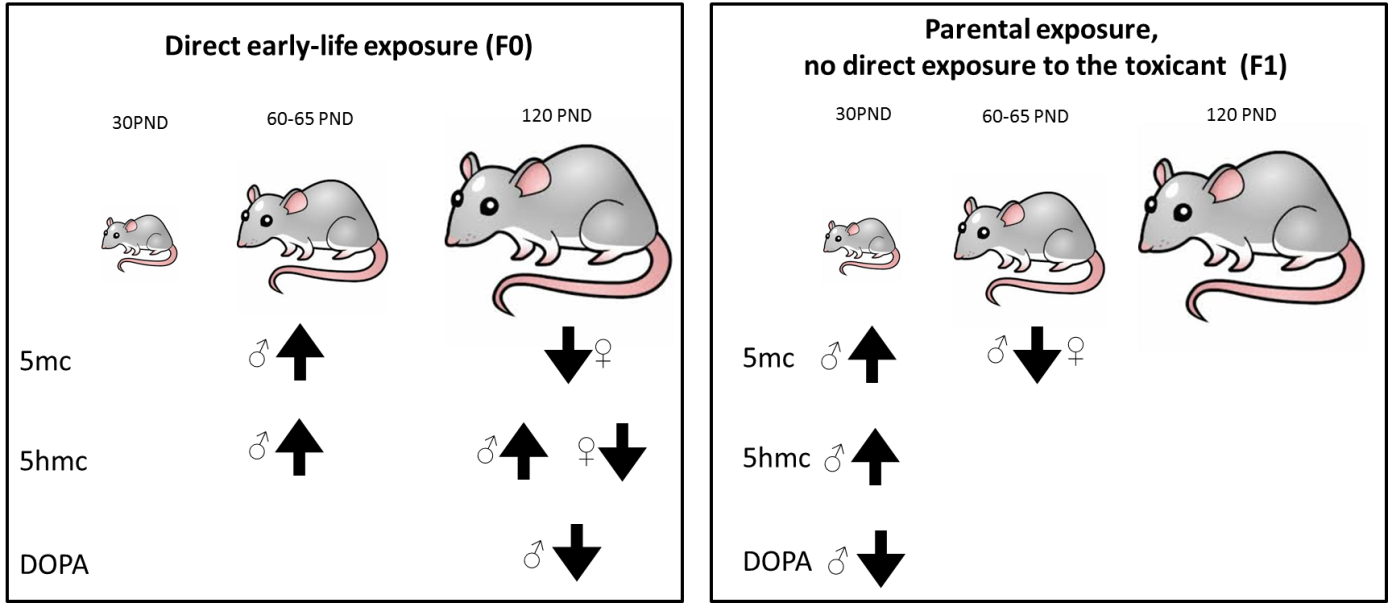
**

**Figure 3 supplementary materials.** Graphical representation of dopamine level, DNA methylation and hydroxymethylation impairments occurring at different ages in early-life treated rats, and their offspring. PND = Post-Natal Days.


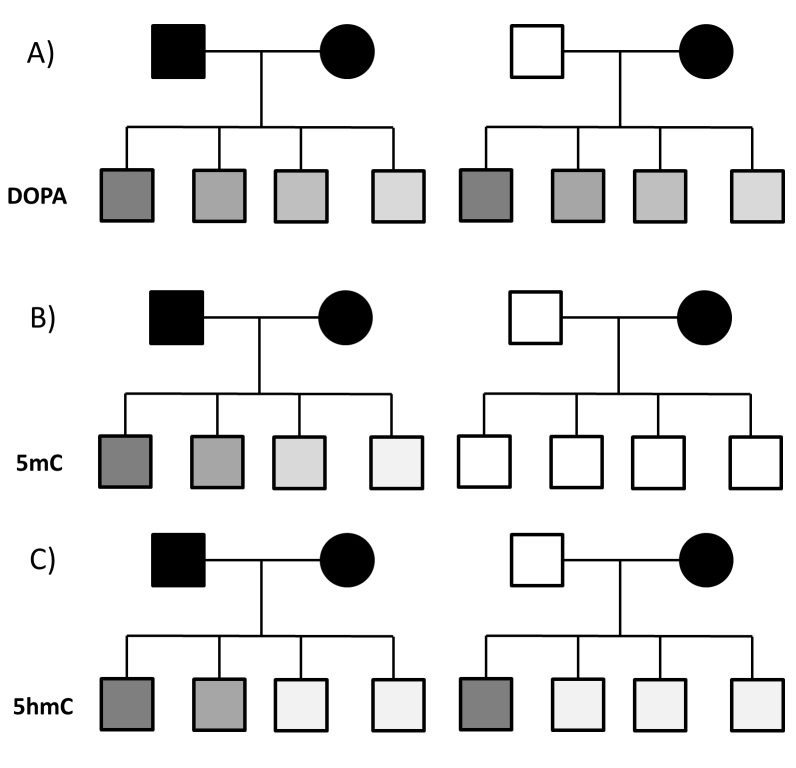


**Figure 4 supplementary materials.** Graphical representation of maternal epigenetic inheritance model for dopamine, DNA methylation and hydroxymethylation phenotypes. Affected family members are illustrated in black, affected off spring are shaded in grey. F1 offspring has been grouped by quartiles of dopamine distributions.
